# The neural dynamics of sensory focus

**DOI:** 10.1038/ncomms9764

**Published:** 2015-11-09

**Authors:** Stephen E. Clarke, André Longtin, Leonard Maler

**Affiliations:** 1 Department of Cellular and Molecular Medicine, University of Ottawa, Ottawa, Ontario, Canada K1N 8M5; 2 Department of Physics, University of Ottawa, Ottawa, Ontario, Canada K1N 6N5; 3 Center for Neural Dynamics, University of Ottawa, Ottawa, Ontario, Canada

## Abstract

Coordinated sensory and motor system activity leads to efficient localization behaviours; but what neural dynamics enable object tracking and what are the underlying coding principles? Here we show that optimized distance estimation from motion-sensitive neurons underlies object tracking performance in weakly electric fish. First, a relationship is presented for determining the distance that maximizes the Fisher information of a neuron's response to object motion. When applied to our data, the theory correctly predicts the distance chosen by an electric fish engaged in a tracking behaviour, which is associated with a bifurcation between tonic and burst modes of spiking. Although object distance, size and velocity alter the neural response, the location of the Fisher information maximum remains invariant, demonstrating that the circuitry must actively adapt to maintain ‘focus' during relative motion.

## Abstract (web summary)

Animals tracking objects can adapt their movements to optimise sensory coding. Using fish that sense objects as perturbations to an electric field, Clarke *et al*. reveal that the optimal object distance maintained by the fish is encoded within the firing properties of electrosensory neurons.

## Graphical abstract

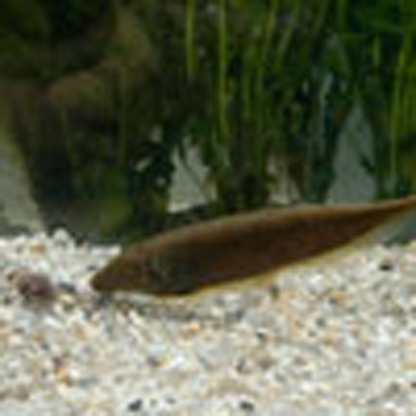

Neural systems that actively engage and track moving objects are faced with two major challenges: determining motion parameters and directing appropriate motor commands to maintain informative sensory input. Behavioural studies on tracking eye movements^1^, fly navigation^2^, electrosensory tracking^3^,^4^ and bat echolocation^5^ have together led to the hypothesis that active sensing can be directed to optimize re-afferent sensory processing. However, the conclusion that active sensing can be executed in a manner that directly benefits neural coding is premature, since it has not been shown that the sensory activity evoked by these motor outputs optimizes neural transmission and thus the resulting behaviour. To assess whether precise tracking performance relies on optimized stimulus estimation, we apply Fisher Information^6^ (*I*_F_) to sequences of action potentials (spikes) recorded from the motion-sensitive responses of electrosensory ON and OFF contrast-coding neurons. By finding where *I*_F_ is maximal along the transverse distance axis (Fig. 1a), we seek to identify where a decoder of ON and OFF cell firing rates may, in theory, achieve the best possible estimation of changes in object position from the observed spiking activity.

The *I*_F_ of a neuron whose spiking activity follows a Poisson distribution, with firing rate function *λ*, can be computed directly as follows (Supplementary Note 2)^6^,


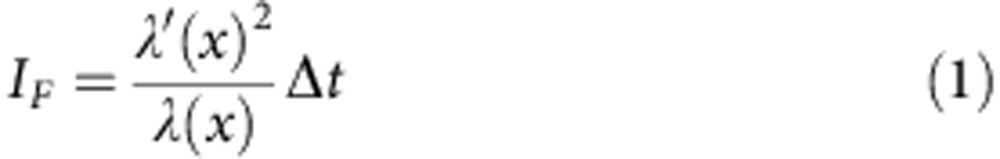


This equation illustrates that the best possible estimate of a stimulus feature (*x*), over an interval of time (Δ*t*), occurs when the square of the derivative of the firing rate with respect to *x*, divided by the firing rate, is maximal. In other words, stimulus-induced changes in firing rate are more readily estimated against lower levels of spiking activity. This estimation principle appears to be reflected in natural behaviour: during echolocation, bats cast their echo-beams off-axis from a target so that the maximum spatial slope, not the peak intensity, of the beam is reflected back to the animal^5^. Despite a weaker signal input, the benefit of this strategy is that small changes in relative distance result in large changes in reflected echo intensity, with putative benefits for motion processing. In an obvious parallel with bats, gymnotiform weakly electric fish track objects at distances that cause relatively small perturbations to their endogenously generated electric field, but with relatively large spatial derivatives (evoking large *λ*′)^7^,^8^,^9^,^10^. We propose that sensing objects at distances that cause weak signals is not simply a consequence of where the slope in the physical signal is maximal. To optimize estimation, a fine balance exists between sensing an object at a distance that evokes a relatively low firing rate in sensory neurons but where relative changes in object distance over time cause large changes in the neural response.

Although the Poisson case is conceptually important and a working approximation under static conditions, we are interested in dynamic stimuli (looming/receding motion) and the assumptions underlying equation (1) may no longer suffice. Therefore, we first sought to examine the statistical nature of pyramidal cell spiking more closely. We show that ON and OFF cell spiking is not a Poisson process under spontaneous or stimulus-driven conditions and then move on to more accurately investigate the idea that optimal estimation in sensory neural networks enables observed tracking behaviours.

## Results

### ON and OFF cell responses to motion

**Figure 1:**
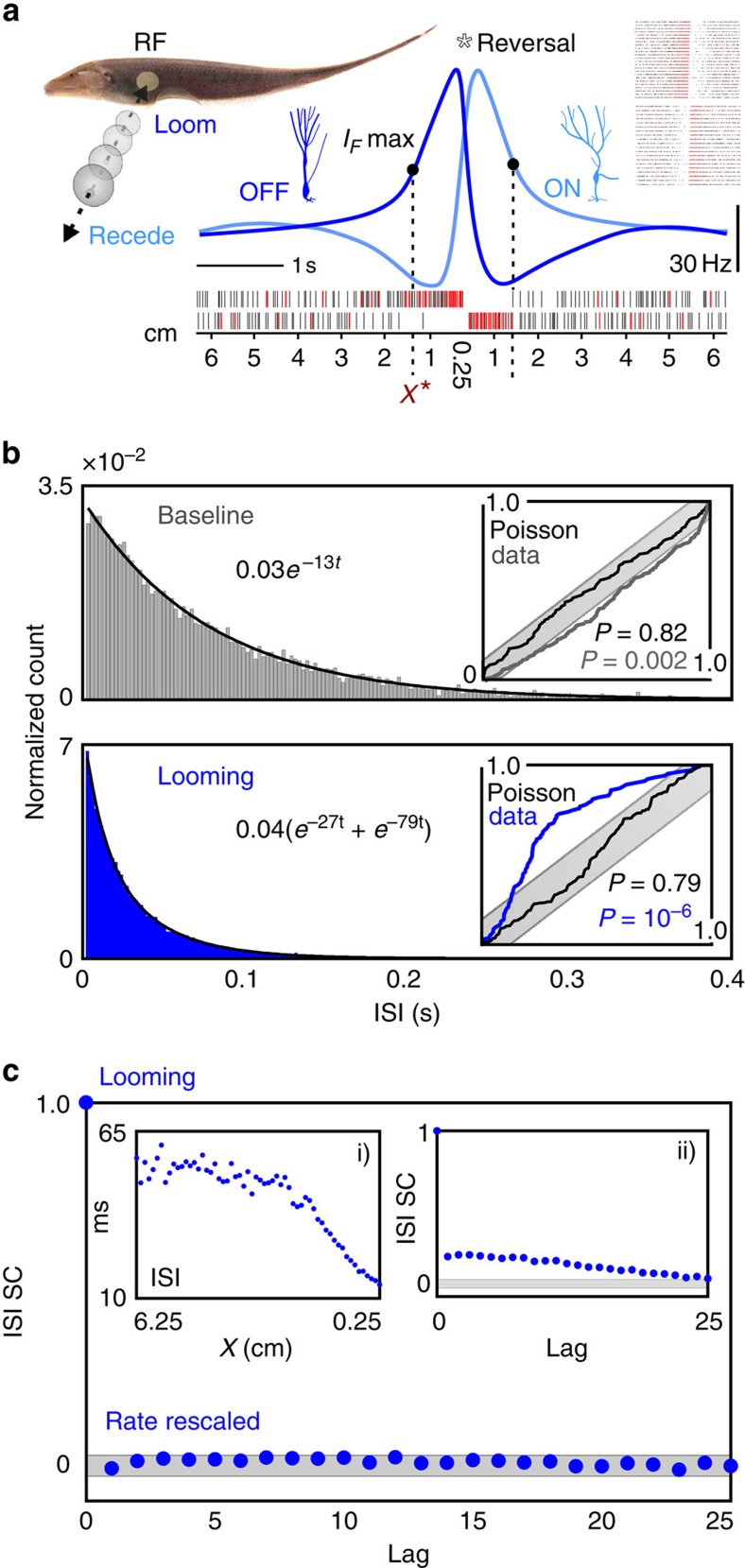
Motion-sensitive ON and OFF cell spiking is not Poisson but is memoryless. (**a**) Recordings were taken from ON and OFF cells during looming and receding motion. The firing rate responses of an ON/OFF cell pair are shown in response to 2 cm s^−1^ motion of a negative contrast stimulus (plastic sphere, diameter *d*=1.21 cm) in their joint receptive field (RF). Examples of the ON and OFF cell spike trains are also shown below the firing rate curves and in the raster plots (upper right), where black lines indicate tonic spiking (ISI >10 ms) and red lines indicate burst discharge (3<ISI≤10 ms). On motion reversal, prominent bursting marks a dramatic switch in the cells' activity; the cell responding to looming motion ceases to discharge, while the opposing cell class is disinhibited and encodes receding motion. The transition from tonic to bursting (looming) and bursting to tonic (receding) closely aligns with the special distance (x*), where *I*_F_ will be shown to be maximal. (**b**) ISI histograms for a population of ON and OFF cells in the absence of a stimulus (baseline) and in response to looming. The best fit exponential(s) for the range of observed ISIs is shown in black. Insets Application of the time-rescaling theorem^13^ removes the stimulus-induced trend from the spiking response, confirming that individual pyramidal cell spiking is not Poisson distributed during stimulation. When the cumulative density of the time-rescaled ISI distribution (ordinate) is plotted against the cumulative density of a uniform distribution (*U*(0, 1); abscissa), the data curve (blue) significantly deviates from what is expected for a Poisson process (the result for a simulated Poisson process of the same duration is shown in black; grey shading denotes 95% confidence intervals; *P* values obtained from a 99% confidence level two-way Kolmogorov–Smirnov test). Sequences of baseline ISIs also deviate significantly from a Poisson process. (**c**) Each ISI is labelled by the spatial position of the object during looming, which yields an averaged non-stationary ISI sequence as a function of object distance, shown in inset (i). Inset (ii) shows that spatiotemporal stimulus correlations are mapped into temporal ISI serial correlations (SC; the correlation coefficient between two ISIs as a function of the lag, or the index number of the recorded sequence). However, after rescaling the ISI sequences, the average serial correlation function demonstrates that spiking can be treated as a renewal process during motion. The grey bands represent 95% confidence intervals associated with the averaged SC function.

*In vivo* extracellular recordings of ON and OFF contrast-coding neurons—located in the primary electrosensory lobe (ELL) of gymnotiform electric fish^11^—were obtained from immobilized *Apteronotidae*, while spherical objects were moved towards (looming) and away (receding) from the animal along the transverse body axis (Fig. 1a). Importantly, our stimulus is a mimic of the type of motion experienced by the electrosense during a behavioural tracking task, the electromotor response^7^,^8^. Brass and plastic spheres were used in our experiments to cast both positive and negative local contrast patterns onto the cutaneous electroreceptors (object contrast is defined by its electrical conductivity relative to the background water). The electroreceptor afferents (EAs) project topographically to ON and OFF cell pairs in the ELL, forming direct excitatory contact with ON cells and indirect contact with the OFF cells through an inhibitory interneuron^11^. As a result, ON cells are selective for increases in stimulus intensity over time and OFF cells are selective for decreases in stimulus intensity, due to the sign inversion. Therefore, motion reversal (switching from looming to receding) changes the sign of the temporal derivative of input intensity, evoking switches in the activity of downstream ON and OFF cells, an event marked by prominent burst spiking^12^ (Fig. 1a). By combining ON and OFF cell outputs, a downstream decoder achieves a bidirectionally symmetric representation of motion. The reader should note that both ON and OFF cells generate looming and receding responses, depending on the sign of the stimulus contrast (only the plastic negative contrast case is presented in Fig. 1a; ON and OFF cell role reversal is observed for brass). In previous work^12^, we show that there is no significant difference between ON and OFF cell responses for looming and receding motion. Therefore, we pooled the data in this paper into looming (ON cell, brass; OFF cell, plastic) and receding (OFF cell, brass; ON cell, plastic) responses. The instantaneous firing rates of individual cell responses were averaged to form ON/OFF cell population firing rates as a function of distance for our eight different stimulus conditions.

### ON and OFF cell spike train statistics

The dynamical transitions between quiescent, tonic and burst spiking states (Fig. 1a, raster plot) impose statistical structure onto the inter-spike interval (ISI) distributions (Fig. 1b). Although the pooled ISI probability densities of ON and OFF cell populations can be described by exponential distributions (with dead time <3 ms for the absolute refractory period), an individual neuron's ISI statistics are not Poisson distributed (Fig. 1b insets; Supplementary Note 1). In particular during motion processing there is strong departure from a Poisson process, serving as an immediate caveat for application of equation (1) to our data. Below we present a generalized formula for locating *I*_F_ maxima, which is inclusive of the spiking distributions characteristic of ON and OFF cells.

In response to looming and receding motion, the average serial correlation of ON and OFF cell ISIs shows that the spiking statistics are strongly non-renewal, that is, the spatiotemporal contrast patterns caused by moving objects produce temporal correlations between successive spikes (Fig. 1c and Supplementary Fig. 1c). To rigorously demonstrate that individual ON and OFF cell spiking is non-Poisson during motion processing, we need to distinguish the intrinsic temporal dynamics of spike generation from those induced by our spatiotemporal stimulus. To this end, we applied the time-rescaling theorem^13^, also used in Fig. 1b (insets), to effectively remove the dependence of the ISIs on the distance of the object (Supplementary Note 1). After removing the stimulus-induced trend in the ISIs, no significant correlation remains, indicative of memoryless (renewal) spiking dynamics over the course of stimulation (see Supplementary Fig. 1b,c for receding motion). In other words, the motion-sensitive responses of ON and OFF cells depend only on the current stimulus value and the timing of the last spike, an important feature of instantaneous rate coding. Therefore, we generalize equation (1) to locate the *I*_F_ maxima of neurons with renewal spiking statistics, free of the restrictive assumption that the observed spiking is described by a Poisson distribution. We expect this result will be relevant for other rate coding systems with irregular, bursty spiking, such as retinal ON and OFF ganglion cells^14^, hippocampal CA1 neurons^15^ and cortical pyramidal neurons^16^.

### Stimulus estimation and Fisher information

**Figure 2:**
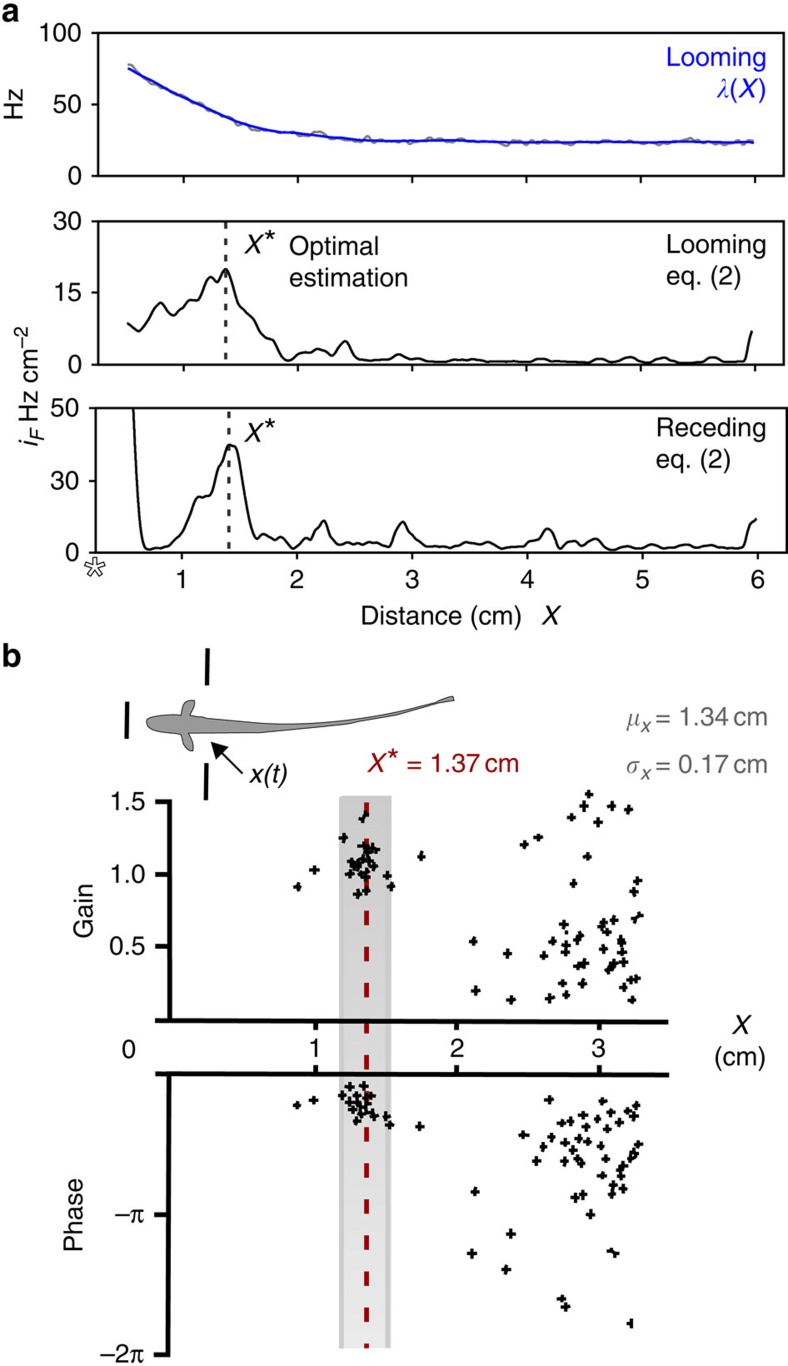
Precise motion tracking aligns with optimal distance estimation. (**a**) Top, in response to the 2 cm s^−1^ looming stimulus (*d*=1.21 cm), the average firing rate of an ON/OFF cell population is plotted against object distance (light grey). The firing rate was then smoothed with a 250 ms moving average filter (blue) to remove small fluctuations, while preserving the stimulus-induced trend. Middle, the nonparametric measure (*i*_F_; equation (2)) is computed from the firing rate and plotted as a function of distance from the leading edge of the stimulus to the skin (*x*(*t*)). For 2 cm s^−1^ motion, this equation is maximal at *x*=1.37 cm, as marked by the dashed line, indicating that this is the point where *I*_F_ is maximal. Repetition of this procedure for speeds of 1, 3 and 4 cm s^−1^, yielded highly similar results with a mean and s.d. of 1.37±0.01 cm, denoted as x* throughout. Bottom, the theory predicts a local *I*_F_ maximum at 1.39 cm for the receding firing rates obtained under continuous motion conditions. The star at the origin indicates motion reversal, and the global maximum in *i*_F_ (*x*<0.5 cm) is due to sudden, sharp transitions from quiescence to high-frequency bursting in the ON and OFF cells. (**b**) The averaged value x* is plotted as a red dashed line over the behavioural electromotor response data of Heiligenberg^8^ (reprinted with permission from the *Journal of Comparative Physiology*). The average gain (1.06±0.14) and phase (−0.68±0.26) were determined for the two data point clusters associated with excellent tracking performance, which occurred at a distance of 1.37±0.11 cm and 1.31±0.13 cm (see Methods section). The composite mean and s.d. of the two clusters (1.34±0.17 cm) is presented as grey shading, showing remarkable agreement with our predicted location for optimal estimation. Note that the other behavioural data points correspond to the fish resting nearly equidistant between the rods (6 cm separation).

An instantaneous population firing rate, which depends on object position (*x*), was determined by averaging individual firing rates obtained from both ON and OFF cells in response to 2 cm s^−1^ looming motion. This function was then substituted into the following formula, which, although not a direct expression for *I*_F_, does locate where *I*_F_ is maximal as a function of *x* (see derivation in Supplementary Note 2):


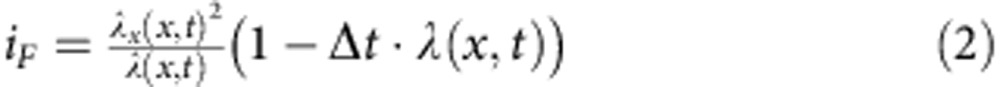


where 
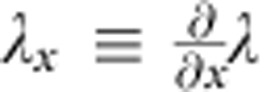
 and Δ*t*=10 ms, a short decoding time window. After repeating this procedure for three more natural looming speeds (1, 3 and 4 cm s^−1^), the mean distance and s.d. for the prominent *I*_F_ maximum was determined to be 1.37±0.01 cm from the fish's body (Fig. 2a; Supplementary Fig. 2; see Methods section for the number of repeats and replicates associated with each case). In this vicinity, the variance associated with the estimation of object distance from the observed spiking activity can achieve its lowest possible value^6^, giving the best possible encoding. For comparison's sake, *I*_F_ was also computed directly using the Poisson formula (1), resulting in poor identification of a maximum (Supplementary Fig. 2).

According to W. Heiligenberg's classic behavioural result^8^, the electromotor response (data reprinted in Fig. 2b), gymnotiform electric fish track sinusoidally moving rods optimally (gain near 1 and phase near 0) while maintaining an average distance of 1.34 cm from the nearest rod. Given the striking agreement between the predicted position at which *I*_F_ is maximal and the position at which the animal best tracks the object, we suggest that the object distance that maximizes the *I*_F_ of a neuron's tuned response is the neural basis of a sensory focal point.

**Figure 3:**
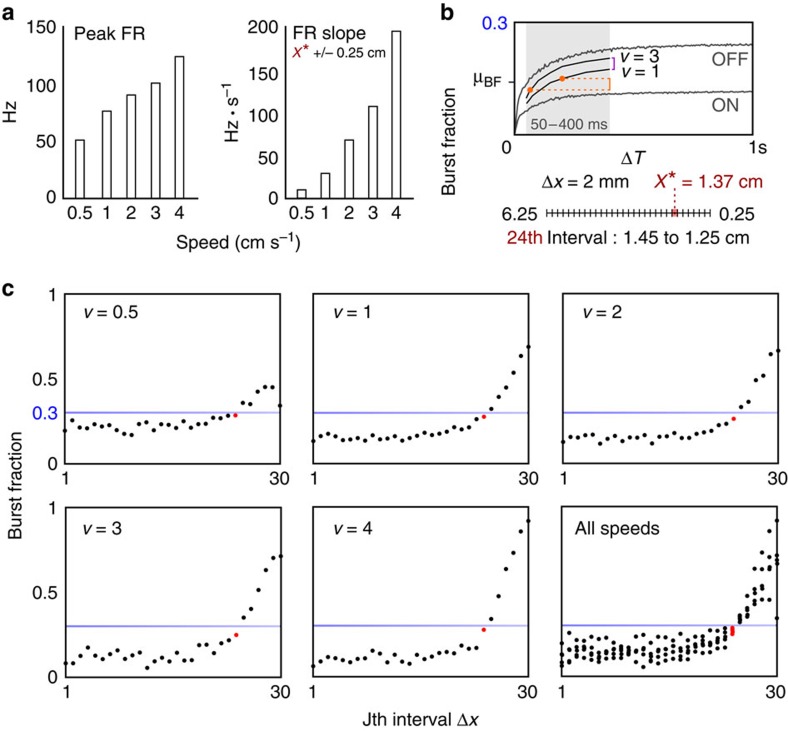
ON and OFF cell responses are speed dependent but sensory focus is speed invariant. (**a**) The peak firing rate and the slope, measured from the line of best fit to the firing rate around the focal point (x*), are strongly sensitive to an object's looming speed. (**b**) BF (number of burst spikes divided by the total number of burst and tonic spikes) was used as a means of measuring how activation of bursting relates to the theoretically identified focal point. The 6 cm distance axis was partitioned into 30 successive 2 mm bins (Δ*x*), each from which BF could be computed. Note the 24th interval, which marks the range of distance 1.25–1.45 cm and contains x*. Depending on the movement speed and the corresponding duration of time (Δ*T*) associated with Δ*x*=2 mm (50–400 ms, for our range of speeds 0.5–4 cm s^−1^) the BF may be underestimated, as shown in the example for ON and OFF cells (grey). For example, the Δ*T* was 200 and 67 ms for *v*=1 and 3 cm s^−1^ data sets, respectively (black), which largely determines an offset between the two stimulus conditions (orange). Additional variability occurs from the different composition of ON and OFF cell responses between the different stimulus condition data sets, which is reflected in an offset of their average BF (purple; diminishing as Δ*T*→0). When comparing between stimulus conditions, we need to be aware of this drift. First, we determined the BF from the first 2 cm of approach where the stimulus has no detectable effect. Next, when examining our BF measure, data sets were aligned to the mean of the entire population (that is, data from all stimulus conditions) determined for the specific time window associated with the interval Δ*x*=2 mm (Supplementary Note 3). This allows for a fair comparison using our simple BF threshold (0.3). (**c**) Averaged population BFs in response to looming stimuli are shown for the 0.5–4 cm s^−1^ cases. For each condition, the interval containing the focal point, marked as a red dot, is found where the proportion of bursting is starting to rise noticeably, but just before the rapid divergence occurring after the BF threshold (0.3±0.005; shaded blue bar). The plot showing all speeds illustrates two interesting facts: variance in the population BF response is squashed in the 24th bin and, from the 25th bin onwards, BF slope reflects motion speed.

ON and OFF cells are sensitive encoders of object speed, reflected in both the peak of *λ*(*x*, *t*) and its slope (Fig. 3a). Despite these strong speed dependencies, the position of maximal *I*_F_ was found to be speed invariant over the range of 1–4 cm s^−1^, using equation (2). However, when applied to the even slower speed of 0.5 cm s^−1^, representative of the electromotor response behaviour, we encountered a methodological issue: the weaker spiking evoked by very slow changes in object position created much more response variability, requiring far greater numbers of trials to obtain sufficiently smooth firing rates. This experimental roadblock to identifying a clear *I*_F_ maximum from a noisy equation (2) inspired us to investigate what aspect of ON and OFF cell spiking activity reflects maximal *I*_F_ *in vivo*, as well as how the electrosensory circuits might decode the responses to produce tracking behaviours for very slow speeds.

### Neuron bursting and invariant sensory focus

Both modelling and experimental studies of pyramidal cells have shown that input signal intensity controls a bifurcation between tonic and burst modes of spiking^17^. Since burst spiking is strongly dependent on input signal slope^18^, is known to be important for feature extraction^19^ and becomes prominent near the focal point (Fig. 1a), we sought to understand how bursting directly contributes to stimulus estimation. In particular, equation (2) shows that the relationship 1-Δ*t*·*λ*(*x*, *t*) scales the Poisson *I*_F_ relationship, suggesting that high-frequency burst spiking (large *λ*) is important for controlling the spike-likelihood within a small coding window (Δ*t*). When a neuron is in the tonic spiking mode, this scale factor, 1-Δ*t*·*λ*(*x*, *t*), is close to one because the chance of observing a spike in the next Δ*t*≤10 ms is low. However, when the cell sharply transitions to the bursting state, there is a dramatic increase in the spike-likelihood and the scale factor drops nearly to 0. Therefore, bursting activity is expected to sharpen local *I*_F_ maxima and enhance focal point acuity (even for very slow motion).

To investigate this idea, the number of burst spikes (3<ISI≤10 ms) divided by the total number of spikes (tonic and burst) was determined within 30 consecutive 2 mm intervals along the distance axis (6 cm trajectory) to compute burst fraction (BF) as a function of object distance (Fig. 3b; Supplementary Note 3). We found a clear increase in the relative proportion of burst spiking in the 24th interval, 1.25–1.45 cm, for looming speeds of 0.5–4 cm s^−1^, occurring very near a data-driven BF threshold of 0.3 (Fig. 3c). Within this interval there is a notable reduction in BF variance. From 1.25 cm onward, dramatic, speed-dependent increases in BF surpass this threshold (Fig. 3c). Note that the 0.3 threshold is a robust measure of burst activation as it sits more than 3 s.d. away from the closely related burst probability of ON and OFF cells under spontaneous conditions (previously reported as ON: 0.25±0.014; OFF: 0.22±0.016) (ref. 20).

**Figure 4:**
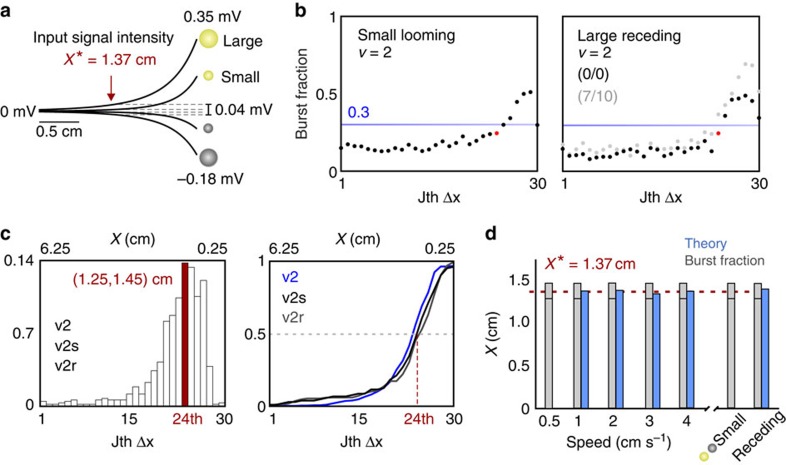
ON and OFF cell responses are intensity dependent but the focal point is invariant. (**a**) Model traces of the electric potential experienced on the skin of the fish for the final 2 cm of looming (or first 2 cm of receding), caused by brass (yellow) and plastic (grey) spheres with small (*d*=0.64 cm) and large (*d*=1.21 cm) diameters^9^. At x* the small spheres create 23% of the stimulus intensity caused by the large spheres of the same material. Furthermore, a plastic sphere generates a signal that is approximately half the intensity of the same-sized brass sphere in absolute value. (**b**) Left, to test whether the focal point is size invariant, looming motion was presented to ON and OFF cells at 2 cm s^−1^ with the smaller spheres. Despite weaker signal intensity, the BF measure, plotted against distance, indicates that the focal point resides in the (1.25–1.45) cm interval. Right, in agreement with the receding data in Fig. 2a, we see a crossing of the BF threshold between the 24th and 25th interval; thus the focal point is invariant to direction for continuous motion (no pauses; 0/0). This point is strengthened by the observation that long pauses (looming, 7 s pause, receding, 10 s pause; 7/10) result in more pronounced bursting in the system and that the focal point shifts to 1.45–1.65 cm according to the BF measure. (**c**) The left panel shows the probability distribution, conditioned on object distance, that a cell in the population will transition to bursting (BF>0.3) and remain in that state. The process was repeated for 2 cm s^−1^ looming (v2), continuous receding (v2r) and the small sphere (v2s), which are pooled together in the histogram—the distribution maximum occurs in the (1.25, 1.45) cm interval. The right panel shows the corresponding cumulative likelihoods of bursting in the population, that is, the fraction of cells in the population that have transitioned to bursting. Despite different input intensities, approximately half of the population is activated around the focal point for each case. (**d**) A summary of the two methods for identifying sensory focus. The capped tops of the grey bars denote (1.25, 1.45) cm. Although methodological limitations prevented successful application of the theory for weaker responses, the BF measure is shown to be a predictor of the distance at which *I*_F_ is maximal.

The match between the BF measure, our theoretical *I*_F_ maximum and the electromotor response behaviour is remarkable, particularly because the size of our stimulus is smaller than the object used by Heiligenberg for the data presented in Fig. 2b (*d*=1.21 cm versus *d*=2 cm). To illustrate the impact of object size and electrical contrast, input intensity curves were generated for our main object size (*d*=1.21 cm), and a significantly smaller object (*d*=0.64 cm) using an empirical model (Fig. 4a)^9^. The intensity of the contrast sensed at the receptive field (RF) of ON and OFF cells scales with the cube of a spherical object's radius. Furthermore, due to their distinct electrical conductivities relative to the surrounding water, a negative plastic stimulus produces a contrast intensity that is half of the intensity produced by the same-sized, positive brass stimulus^9^,^12^. These observations suggest that the location where *I*_F_ attains a local maximum is also intensity invariant, consistent with recorded behavioural performance for a range of object sizes^8^. To test this hypothesis directly, we presented 2 cm s^−1^ looming motion using the smaller stimulus (*d*=0.64 cm), which is a lower bound for object size and electromotor response performance^8^. At the location of the *I*_F_ maximum (x*=1.37 cm), the signal intensity for the smaller sphere is 23% that of the larger sphere. Yet, when using our BF measure we found that the focal point was still located in the 1.25–1.45 cm range (Fig. 4b, left; Supplementary Table 1).

Despite a bidirectional physical symmetry with the traces of Fig. 4a, receding stimuli evoke a skew in the EAs firing rate responses compared with the looming responses, which is caused by directionally selective spike-rate adaptation^12^. This asymmetric EA response causes an apparent disconnect between the feedforward sensory input, pronounced burst spiking and switches in ON and OFF cell coding^12^. Yet, the location of the *I*_F_ maximum is preserved in the population (Fig. 2a, bottom; Fig. 4b, right), provided continuous motion occurs, as in the electromotor response protocol. Interestingly, if long pauses (looming, 7 s pause, receding and then 10 s pause) are inserted, there is no longer a notable reduction of the receding burst response. These pauses create a static object distance and are therefore unnatural for tracking behaviours; in reality continuously varying relative motion is experienced by the network. Under the pause circumstances the *I*_F_ maximum is now found at 1.72 cm according to the theoretical approach. The BF measure also shifts further from the body to the 1.45–1.65 cm distance interval (Fig. 4b, right), which strongly supports the idea that burst onset is a major determinant of the focal point location (Supplementary Fig. 3).

Motion direction- and size-invariance are particularly surprising features of the focal point. An additional population-coding perspective is provided in Fig. 4c (left) for our 2-cm s^−1^ velocity cases (default-sized looming, default-sized receding and small looming), which shows that the maximum probability for a neuron in the population to transition to bursting occurs in the 24th interval (1.25, 1.45) cm. This peak corresponds to the cumulative probability that approximately one half of the neurons in the stimulated population have transitioned to bursting Fig. 4c (right) and could be viewed as a tipping point between two population activity states. Remarkably, these population distributions are preserved for both directions of motion and for markedly different stimulus intensities. The close agreement between the focal point location predicted by Fisher Information and BF (Fig. 4d; Supplementary Table 1) is even more convincing in light of these burst-probability distributions.

## Discussion

We have demonstrated that the accuracy of the information conveyed by ON and OFF cells about the location of a transversely moving object is maximal between 1.3 and 1.4 cm from the fish's skin. This location is independent of the nature of the object (positive or negative contrast), its direction of motion, its speed and its size. Importantly, this location is precisely the one chosen by an electric fish as it tracks a transversely moving object in a classic behavioural paradigm. The methods we used to compute the location of the *I*_F_ maximum are sophisticated, and, at first, it was not clear how a neural network could actually implement a focal point. ON and OFF cells burst in response to transverse motion and we discovered that a burst detector could, in principle, accurately read out the theoretically defined optimal location. Detailed studies have elucidated the ON/OFF cell burst mechanism^21^,^22^,^23^ and identified numerous potential modulators of bursting^24^,^25^. As discussed below, these may be the cellular and network bases by which the burst mechanism is controlled to give rise to an invariant focal point.

The fact that a stable *I*_F_ maximum was maintained for both ON and OFF cell types under many stimulus conditions implies that the ELL network must actively adapt to the electrosensory input to dynamically control gain and burst onset in the ON and OFF cell population. This is a unique example of Barlow's efficient coding hypothesis^26^, which holds that sensory systems should transmit information optimally by adapting to natural stimuli. However, the manner by which electrosensory networks adapt to different objects and control focal point stability is unknown. The observed speed invariance of the *I*_F_ maximum to looming motion may follow directly from two phenomena: a special computation performed by the EAs and the intrinsic bursting properties of ON and OFF cells. First, a scale-free (power law) form of spike-rate adaptation transforms EA firing rates to accurately encode changes in looming object distance, regardless of the stimulus timescales (that is, looming speed)^27^. Second, both modelling and experimental studies of ELL pyramidal cells have shown that feedforward input intensity controls a saddle-node bifurcation between tonic and burst modes of spiking^16^. As such, object distance will be directly encoded by the EA firing rates independent of the object's looming speed^27^ and could trigger a burst bifurcation in the downstream ON and OFF cells at a specific position, defined by the biophysical characteristics of the network.

Although the EAs are able to remove speed-dependent effects from their firing rates, the magnitude of their responses is strongly sensitive to different object sizes^27^. Therefore, assuming a hard-wired network response, it is reasonable to expect that the smaller object would trigger ON or OFF cell bursting much closer to the sensory surface, whereas larger objects would cause bursting to occur at distances further from the skin. However, this is not the case. The observed invariance of the *I*_F_ maximum to object size firmly demonstrates that the burst onset can't simply trigger off of the feedforward input. As a further example, our analysis pooled ON cell responses to looming brass spheres with OFF cell responses to looming plastic spheres since they are indistinguishable^12^. Likewise, receding responses of ON cells to plastic and OFF cells to brass were pooled. This simple fact is actually surprising given the stimulus intensity of a brass object is twice greater than the same-sized object made of plastic (Fig. 4a). Clearly multiplicative^24^ and divisive^28^ gain control must be implemented by the network to influence bursting and stabilize the focal point. An understanding of such flexible gain control for dynamic stimuli is an exciting challenge for future work. Note that adjusting the overall gain based on object size and conductivity would allow for effective operation of the hypothesized speed invariance mechanism described above.

Receding motion further challenges our understanding of how burst spiking is controlled in the network. Despite the fact that the feedforward EA response to looming is considerably different than the receding response, downstream ON and OFF cell receding firing rates are nearly mirror-symmetrical versions of the looming responses^12^ and the focal point is preserved for continuous motion. In this scenario, the dynamical states are visited in the reverse order, but with the same result, illustrating that dynamical transitions can optimize signal estimation regardless of their temporal sequence (burst to quiescence or vice versa). Further modelling and experiments on balanced excitatory and inhibitory feedback pathways will be required to directly connect dynamic physiological control of the tonic-burst spiking bifurcation to both the observed behavioural responses and optimized stimulus estimation. A clue to potential mechanisms may lie in the robustness of the spiking activity patterns of single neurons in response to concerted changes of multiple conductances induced by neuromodulators^29^. The invariant location of the *I*_F_ maximum would presumably require coordinated changes in synaptic conductances of the ELL network to control the location of the burst bifurcation in the face of varying electrosensory input.

The ON and OFF cells project to the midbrain (torus semicircularis) where there is an explosion in the number of cell types, indicative of early feature extraction^11^. Previously, directionally selective cells have been identified in the torus semicircularis that are highly sensitive to longitudinal motion; however, further work is required to characterize torus semicircularis cells that are selective for the transverse looming and receding motion. It has previously been suggested that the burst and tonic spikes of electrosensory ON and OFF cell spike trains can be segregated by facilitating and depressing synapses, respectively^30^; both forms of plasticity have been observed in torus semicircularis neurons^31^,^32^. Therefore, we propose that midbrain networks can processes tonic and burst spiking in parallel to compute BF and identify the location of optimized distance estimation.

Extensive neuroanatomical studies^11^ have demonstrated that a strong projection from torus semicircularis to the tectum is the only route from the electrosensory periphery to the motor system. The electromotor response behaviour presumably uses this pathway during active sensing; where stimulation of the EAs leads to motor outputs that simultaneously influence the re-afferent sensory processing. We hypothesize that sensory focus is not an open-loop, cascade-like computation but rather a closed sensorimotor loop that arises during active sensing behaviour—the fish finds a location that maximizes the information from its electrosensory input and then uses this optimal input to guide subsequent motor outputs.

The simultaneous activity of sensory and motor systems during tracking behaviour is expected to preserve the relative distance as follows. The animal should adjust its position such that the object distance triggers bursting in approximately half of the stimulated population (Fig. 4c). On the cusp of transitions between different dynamical states, a downstream decoder can optimally estimate object position based on the evoked spiking activity of ON and OFF pyramidal cell populations. In this region of space, better estimates of object distance are expected to permit more accurate estimates of speed, which are reflected in the temporal slope of the pyramidal cell firing rates (Fig. 3a). For a given object contrast, increases and decreases in signal intensity indicate changes in the relative distance and are expected to dictate appropriate orienting behaviours. On changes in motion direction, the prompt bursting of either the ON or OFF cell populations appears very well suited to guide compensatory motor commands to maintain object focus.

## Methods

### Surgical procedure

Surgery was performed on adult male and female gymnotiform fish, *Apteronotus leptorhynchus* (imported from natural habitats in South America), to expose the caudal cerebellum overlying the ELL. All surgical and experimental procedures were reviewed and approved by the Animal Care Committee at the University of Ottawa. Immediately following surgery, fish were immobilized with an injection of the paralytic pancuronium bromide (0.2% w/v), which has no effect on the neurogenic discharge of the electric organ that produces the fish's electric field (EOD)—the basis of the electrosense. The animal was then transferred into a large tank of water (27 °C; electrical conductivity between 100–150 μS cm^−1^) and a custom holder was used to stabilize the head during recordings. The tails were gently tethered in position with thread to avoid any potential displacement of the body due to the small hydro-mechanical effects caused by looming/receding motion. All fish were monitored for signs of stress and allowed to acclimatize before commencing stimulation protocols.

### Neurophysiology

Extracellular recordings were taken from pyramidal cells of the centrolateral map of the ELL^11^. This map was chosen because its neurons respond strongly to object motion and have fairly large, easy-to-locate RFs^33^. Recordings were obtained from cells whose RFs were located 30–65% along the rostral-caudal body axis of the animal, as this region provides the flattest body surface and EOD isopotentials with low curvature that lay perpendicular to the looming/receding stimulus trajectories. Likewise, since the body curves away from the ‘sensory plane' on the belly and back, distance is harder to control for and only cells whose RFs were in the 25–75% range on the dorsal ventral axis were used. These restrictions were to ensure a consistent electric image that was not warped by body geometry or the field boundary effects occurring at the interface of tank water and air. Importantly, this range of the body surface includes the location where the gymnotiform fish *Eigenmannia virescens* align themselves during the electromotor response behaviour^7^,^8^. After finding a cell's RF centre using a local stimulus dipole, we classified it as ON or OFF based on its response to step increases and decreases in the local field potential. We then mapped out the RF centres, which yielded spatial spreads consistent with anatomical estimates for the centrolateral map^33^. The baseline firing rates of the recorded ON and OFF pyramidal cells (5.4–25.9 Hz; *N*_E_=15, *N*_I_=16) demonstrate that they are the superficial and intermediate types^11^. In some cases, we were able to simultaneously record from ON/OFF cell pairs and directly compare their differential responses to object motion. A particularly nice example of one such pair is displayed in Fig. 1a.

Using our total population of 31 cells (Supplementary Fig. 1a), we computed a representative s.d. as *σ*=17.06 × 0.95=16.21 Hz, to determine a sample size (*n*) as


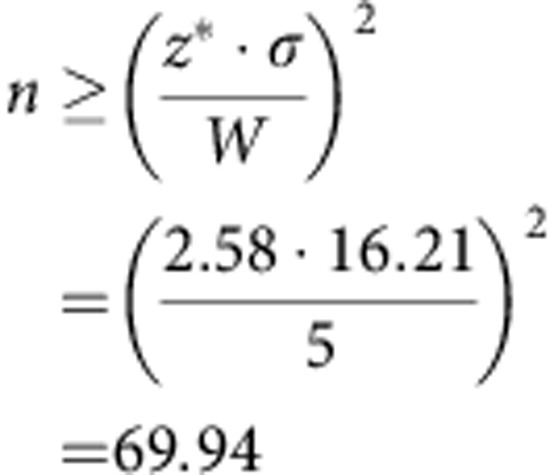


for a 99% confidence level (z*=2.58) on a confidence interval width (*W*) of ±5 Hz. From this simple calculation, we see that we need at least 70 trials. However, when choosing a sample size for estimating mean firing rates in a population of neurons by averaging repeated trials, we need to consider the following. It is not clear that the use of the Z-statistic, which is related to the normal distribution, is correct since we are sampling different classes of ON and OFF cells whose spiking statistics are inhomogeneous and whose sampling cannot be treated as independent and identically distributed. There are a number of other complicating factors in determining our sample size, including the statistically non-stationary responses of ELL pyramidal cells to motion and the fact that there are two sources of variance: the variance of single cell's response to repeated trials, as well as the variance in the population spiking statistics. In the above calculation, we chose to use spike train statistics in the absence of a stimulus—although the variance grows larger when the stimulus is present, there are also more spikes present, which, when averaged, yield smoother firing rate estimates compared with baseline levels. The degree of smoothing is further influenced by the length of the smoothing kernel (although it was chosen to be a minimal 10 ms in the work presented here). Without a well-defined formula to compute an exact required sample size, we turned to practicality, averaging enough trials to get sufficiently smooth firing rates to readily discern the stimulus-induced response and address our hypotheses. We well exceeded the calculated population size for all of our data conditions, with the exception of 0.5 cm s^−1^, which takes 24 s to do a single trial, greatly diminishing the amount of data that can be collected. Our largest sample, *N*=463, yields 99% confidence on an interval of ±1.94 Hz. The number of replicates (*N*_1_) and repeats (*N*_2_) for each of our stimulus conditions are listed here and were used for both the theory approach in Fig. 2 and the BF measures of Figs 3 and 4. For the large looming sphere: 0.5 cm s^−1^, *N*_1_=9, *N*_2_=73; 1 cm s^−1^, *N*_1_=23, *N*_2_=255; 2 cm s^−1^, *N*_1_=31, *N*_2_=463; 3 cm s^−1^, *N*_1_=13, *N*_2_=120; 4 cm s^−1^, *N*_1_=15, *N*_2_=166. For the large receding sphere (2 cm s^−1^): continuous (0/0), *N*_1_=14, *N*_2_=168; discontinuous (7/10), *N*_1_=13, *N*_2_=147. Finally, the small looming sphere (2 cm s^−1^): *N*_1_=8, *N*_2_=104.

According to cell type, a plastic or brass sphere was connected to an electromechanical positioner, which was pre-programmed for the appropriate motion sequence and initiated by outputs from our data acquisition software (Spike2 v7.03; Cambridge Electronic Designs). The selected sphere was aligned with the cell's RF centre along the transverse (lateral) body axis and placed at the initial position, *x*_0_=0.25 cm from the skin. Note that mal-alignment with a degree of ±0.05 cm error was unavoidable when setting *x*_0_ by using 0.1 cm gradations to measure the position of the sphere's leading edge relative to the fish's body. Slight mal-alignment of the sphere's centre with the RF center is another small, but unavoidable source of error. For our large sphere, *d*=1.21 cm, the effect of mal-alignment on the electric image is negligible since the sphere easily saturated the RF. The sphere was then withdrawn and motion was initiated a few moments later. Stimulation consisted of consecutive repetitions of looming and receding sequences at a speed of 2 cm s^−1^, chosen as an intermediate value from studies of gymnotiform locomotion^7^,^8^,^10^. This stimulation protocol was then repeated with a sphere creating an electric image with the opposite contrast, as well as for varying speeds (0.5–4 cm s^−1^). Acceleration at the beginning and end of constant velocity looming/receding motion profiles was set to 150 cm s^−2^, so periods of non-uniform velocity were negligible (<0.05 cm in all cases). For 13 of the 31 pyramidal cells used in this study, the same loom/recede sequence was repeated with a 7 s pause at the skin before receding to the initial position and a 10 s pause before the next trial (as performed in a previous study^12^). Typically 10 trials were obtained per cell, but for a few cells it was as many as 25. Population-averaged firing rates were computed by convolving the individually recorded spike trains with a minimal, 10 ms exponential kernel and then averaging across all trials and all cells to obtain smooth response curves for each stimulus condition. The example firing rate curves displayed in Fig. 1a were generated using a smoothing technique employed in previous work^12^ (see reference within), however, this technique was not used for the analysis since the algorithm assumes that spiking can be treated as a Poisson process. Note that convolution of the spike train with a smoothing kernel results in a sharp rounding down of the firing rate at the very ends of the file, where the recorded spiking abruptly stops. As can be seen in Fig. 2, as well as in Supplementary Figs 2 and 3, we routinely clip 0.25 cm from one (receding) or both (looming) edges of the recordings to avoid this false curvature that causes large spurious jumps in equations (1) and (2). The firing rates were then further smoothed with a short-duration moving average filter of 250 ms, as shown in Fig. 2a (top). Notice that the smooth curve (blue) is still a very strong representation of the underlying firing rate. The curves resulting from equations (1) and (2) were then smoothed once more with a 100 ms moving average filter, enabling us to identify very clear maxima.

We collected data with two conventional sphere sizes, used previously by Chen *et al*.^9^ in detailed models of the electrosensory image: our default sphere, an intermediate size from Heiligenberg's studies with a diameter (*d*) of 1.21 cm, and a substantially smaller sphere (*d*=0.64 cm), which was used as an object size control. Heiligenberg^8^ (Fig. 10 within) showed that strong gain in the electromotor response performance begins to deteriorate for *d*≈0.8 cm and significantly so for the object diameters <0.6 cm, thus the small stimulus is expected to represent a meaningful lower bound for network processing during motion tracking. The smaller sphere creates very localized changes in the electric potential; in many cases it seemed we were on the edge of the RF, partially stimulating the surround as evidenced by the occasional total lack of response, only to see it appear again. For this reason, we only used data in which a clear, strong response was observed, omitting cases in which the firing rate was inconsistent from trial-to-trial and dropped dramatically on some trials.

In general, data trials were only excluded when external noise was present in a recording or if a cell showed pathological signs of activity, such as hyper-excitability or lack of response to the stimulus. This may occur due to cell fatigue, damage or anoxic stress.

All data analyses were performed using custom Matlab scripts, which can be made available to those interested on request to the corresponding author. Application of the time-rescaling theorem in particular is the most relevant; the remaining code is straight forward or intended to interact with our specific recording software.

### Estimation of tracking distance from behavioural data

Heiligenberg's original electromotor response data^8^, presented in Fig. 2b of the main text, was imported into Matlab to identify the positions of the individual data points in the image relative to the axes; this allowed us to compute sample statistics for the obvious clusters, which were not provided explicitly in his paper. This was accomplished with open source code that can be found on the Matlab Central website (ReversePlot 2009, Jordi Palacin. All rights reserved.) For the top half of the plot (gain versus distance), this method yielded a mean and s.d. of 1.06±0.14 cm for the gain, occurring at distance of 1.37±0.11 cm. Repeating the analysis for the bottom half of the plot, we found a phase of −0.68±0.26 radians, occurring at a distance of 1.31±0.13 cm. The average of these two mean distances and their corresponding composite s.d. is 1.34±0.17 cm, which is overlaid as grey shading onto the behavioural data.

It is important to note that the electromotor response data sets were obtained using the weakly electric gymnotiform fish *E. virescens*. Like the closely related gymnotiform *A. leptorhynchus*, they produce a continuous, high-frequency EOD and have nearly identical electrosensory afferents and hindbrain circuitry^34^. Unfortunately, their electric organ is myogenic and the pancuronium bromide injections used for immobilization blocks the electric organ discharge. A mimic EOD can be generated by mounting an electric dipole onto the animal, with one end in the mouth and the other looped around the tail. Although this is suitable for studying broad global stimuli relating to social interactions with conspecifics, the resulting EODs are highly artificial and do not reflect the local spatial aspects of the field *in vivo*. Despite being useful as a behavioural species, *Eigenmannia* is not well suited to our neurophysiology protocols.

## Additional information

**How to cite this article:** Clarke, S. E. *et al*. The neural dynamics of sensory focus. *Nat. Commun*. 6:8764 doi: 10.1038/ncomms9764 (2015).

## Supplementary Material

Supplementary Information Supplementary Figures 1-3, Supplementary Table 1, Supplementary Notes 1-3 and Supplementary References
