# Supplementary material for: The neural dynamics of sensory focus
Source: Nat Commun. 2015 Nov 9;6:8764. doi: 10.1038/ncomms9764 (PMC4659932; doi:10.1038/ncomms9764)
Supplement: Supplementary Information — Supplementary Figures 1-3, Supplementary Table 1, Supplementary Notes 1-3 and Supplementary References [file ncomms9764-s1.pdf]

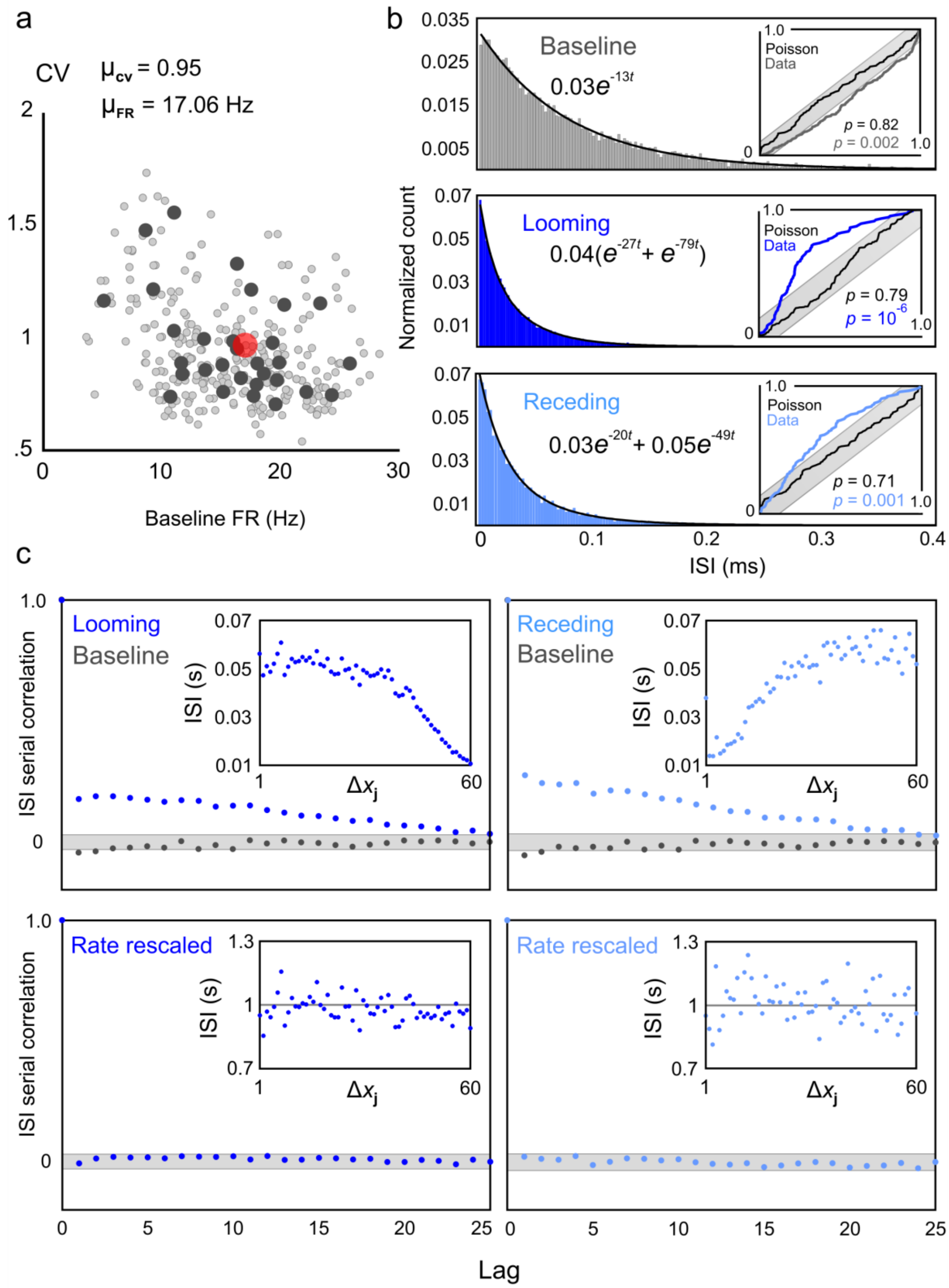

Supplementary Figure 1 **ON and OFF cell statistics during the absence and presence of a motion stimulus** a) Our population average CV (red dot) is plotted against baseline firing rate, along with the average CV values of individual ON and OFF cells used in this study (dark grey;

N = 31) and an additional 200 values (light grey) determined from 6 second clips of spontaneous spiking, randomly sampled from the population. b) Best fit exponential (baseline) or sum of exponentials (looming and receding) are shown for the population ISI histograms, where the bin size was chosen to minimize a cost function between the sample histogram and the unknown underlying probability density[1]. Note that here, and in Fig. 1a, the fit neglected the absence of ISIs smaller than the 3 ms refractory period. The discrete time-rescaling theorem was applied to test whether the underlying spiking could be described as a Poisson process – clearly the null hypothesis is rejected (both visually and by the *p*-values associated with the 99% confidence level, two-way Kolmogorov-Smirnov test. In particular, receding data is included for comparison with the looming and baseline results shown here and in Fig. 1a. c) Our looming and receding stimuli impart strong, positive temporal correlations to the successive ISIs. The insets show the population averaged ISIs within 60 intervals (Δx = 1 mm) along the distance axis. This stimulus-induced trend is reflected in the ISI serial correlation (SC) functions. Many pyramidal cells show a weak negative correlation in the absence of stimulation, reflected in the population averaged SC (grey bands represent 95% confidence intervals in all plots). The rescaled ISIs during looming and receding motion can be treated as a renewal point process with unit rate.

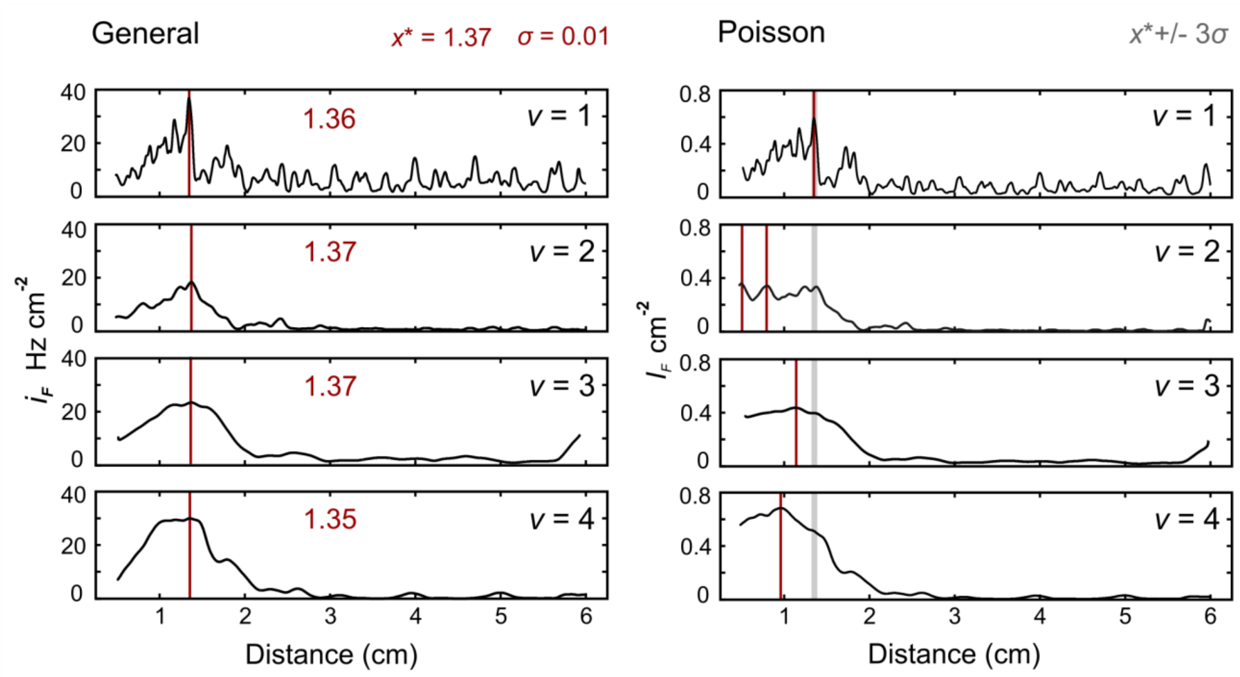

Supplementary Figure 2 **Identification of maximal I_F with a nonparametric versus Poisson relationship** The derived measure for locating the local I_F maxima (i_F, Eq. [2]) was applied to the 1 - 4 cm/s looming population data, yielding a mean and standard deviation of 1.37 +/- 0.01 cm, presented as x^* in the main text figures. The Poisson case (Eq. [1]) is noticeably worse at identifying a consistent maximum; the grey shading in the Poisson panels show three standard deviations about x^*. A time interval of $\Delta t = 10$ ms was used for v = 1 and 2 cm/s, whereas $\Delta t = 8$ ms was used for v = 3 and 4 cm/s since the peak firing rates exceeded 100 Hz in these cases, causing Eq. [2] to become negative (Fig. 3a; Supplementary Note 2). The choice of 8 versus 10 ms has a negligible effect on the location of maximal I_F, where the spiking rates are still relatively low: the maximum shifts to and 1.39 cm for v = 3 cm/s and 1.37 cm for v = 4 cm/s. We chose to use the values for $\Delta t = 8$ ms, since it respects the conditions of the theory, while giving even tighter estimates. Importantly, this choice does not impact the paper's conclusions.

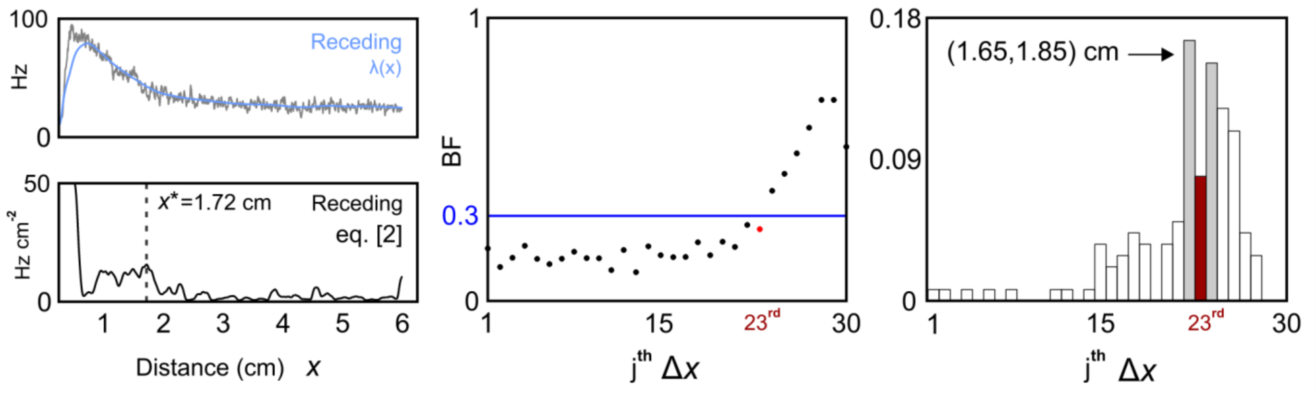

Supplementary Figure 3 **Discontinuous motion shifts the focal point for receding** When long pauses are inserted between looming and receding trials (looming, 7 second pause, receding 10 second pause, repeat) the theory (left) predicts a focal point at 1.72 cm for receding motion. This is reflected in the shifted burst fraction measure to the (1.45, 1.65) cm interval (center) and the probability of a neuron in the population transitioning to the burst state as a function of object distance (right). Notice that this probability distribution is highly reminiscent of Fig. 4c in the main text, except there is now another pronounced maximum in the 22nd interval (1.65, 1.85) cm, reflecting the extra bursty responses not present in the continuous motion trials.

BF I_F measure	$\Delta T_1 = 0.1/v$	$\Delta T_2 = 0.15/v$	$\Delta T_3 = 0.2/v$	$\bigcap_i \Delta x_i$	Theory
v = 0.5 cm/s	49/60	32/40	24/30	[1.33, 1.35]	-
v = 1 cm/s	49/60	33/40*	24/30	[1.25, 1.3]	1.36
v = 2 cm/s	50/60*	32/40	24/30	[1.3, 1.45]	1.37
v = 3 cm/s	51/60*	32/40	24/30	[1.3, 1.45]	1.37
v = 4 cm/s	48/60	33/40*	24/30	[1.35, 1.45]	1.35
Small (2 cm/s)	51/60*	33/40	24/30	[1.25, 1.3]	-
Recede 0/0 (2 cm/s)	49/60*	32/40	24/30	[1.3, 1.45]	1.39

Supplementary Table 1 **Assessing BF results for varying window lengths** To assess how window length impacted our findings, and to determine which window length was most congruent with the theory, BFs were computed as a function of object distance for three fixed spatial intervals (Δx_1 = 1 mm, Δx_2 = 1.5 mm and Δx_3 = 2 mm). Each entry in the table records the distance interval found just before the BF threshold (0.3) as a ratio of the total number of intervals in which the 6 cm trajectory is subdivided. To avoid underestimating the BF (Fig. 3b), and to account for some discrepancy between the population subsets used for each stimulus condition, the mean was measured for the first third (2 cm) of the approach, where the stimulus is undetectable, and then adjusted to match the mean population BF for its specific time window, $\Delta T = \Delta x / |v|$, determined from all 31 cells used in the study (Methods). Determining where the BF exceeds our threshold for these three different spatial intervals allowed us to compute their intersection and narrow down the predicted location of the I_F maxima (x^*) for each condition, for comparison with the theory. For the cases in which Eq. [2] was applied (1-4 cm/s looming and 2 cm/s receding), we found that the BF value lying just before the hard threshold of 0.3 contains the theoretically identified focal point consistently. The burst fraction intervals produced identical results for the 0.5 cm/s looming stimulus, which is in agreement with strong electromotor response behavior at slow speeds[8]. For the finer resolution (1 and 1.5 mm), the time windows grow shorter and underestimation appears to weaken the fidelity of our BF measure. In particular, the instances marked with a red star were omitted from the intersection operation as

they generate the null set and stand at odds with the theory and behavior. Since 1 and 1.5 mm appear less reliable, we chose 2 mm as our distance interval for the BF analysis in Figs. 3 and 4 of the main text. In the case of the small sphere, both 1 and 1.5 mm predict that the sphere's focal point is actually a bit closer to the skin but still in the range [1.25, 1.35] cm (discrediting the 1 mm interval as per above), just like the v = 1 cm/s case. The results for the 1 and 1.5 mm interval may be due to chance, due to underestimation of the BF, to the potential under-stimulation due to more difficult RF alignment (see Methods), or to the limitations of using a hard threshold as opposed to more sophisticated synaptic decoding, hypothesized to occur in vivo. However, the result may also be meaningful since the d = 0.64 cm object is the absolute smallest size for which electromotor motion tracking was observed – gymnotiform fish appear uninterested in or unable to continuously track smaller inanimate objects. Therefore, the small object's focal point may be beginning to shift, accompanied by the diminishing behavioral response. Whether a I_F maximum is still important for even weaker stimuli is a question for future work.

Supplementary Note 1 ON/OFF cell spiking is memoryless during motion processing

ON and OFF pyramidal cell spiking is highly irregular, which can be seen in the raster plots of Fig. 1a. Supplementary Fig. 1a shows a scatterplot of the coefficient of variation (CV) of each cell's ISIs as a function of their baseline firing rate. Note the mean of the population (0.95), with many cells having a CV near one. This suggests that the spiking of many ON and OFF cells might be reasonably described as a Poisson process (with dead time, that is, no spiking during the action potential refractory period < 3 ms). In the absence of a stimulus (or baseline conditions), histograms of inter-spike intervals (ISIs) recorded from the entire population (Supplementary Fig. 1a) were pooled for ON and OFF cells and are very nearly fit by exponential distributions on the range of observed ISI times, (0.003,0.62) seconds (Figs. 1a, Supplementary Fig. 1b). These facts suggest that treating the baseline spiking of a pyramidal cell population as a Poisson processes is reasonable.

However, the individual ON and OFF cells have a CV that ranges from as low as 0.5 to just over 1.5, indicating that spiking is non-Poisson for many ON and OFF pyramidal cells. Pyramidal cell ISIs may actually arise from a Gamma process[2] (with a scale parameter k that varies from cell to cell); this would account for the observed range of CVs, given by $(\sqrt{k}\mu_{ISI})^{-1}$, where μ_{ISI} is the ISI sample mean. Despite this fact, the best fits of Gamma distributions were unsatisfying. Furthermore, at baseline, the average serial correlation function between successive ISIs shows a weak, yet significant negative correlations(Supplementary Fig. 1c), violating the renewal assumption that is implicit to both Poisson and Gamma processes. It thus appears that baseline pyramidal cell spiking in the ELL, as a whole, cannot be described by common parametric distributions. Therefore, we sought to use survival analysis[2] to develop a simple

connection between our empirical firing rates and Fisher's information, as previously done for Poisson spiking neurons (Eq. [1])[3] .

During stimulus presentation, population ISI distributions show compound exponential behavior (Supplementary Fig. 1b). To demonstrate that individual pyramidal cell spiking is also non-Poisson during stimulation, we applied the discrete time-rescaling theorem[4, 5]. This technique effectively detrends the spiking response. Instead of using the stimulus dependent spike likelihood obtained from a model distribution of spiking (e.g. Poisson or Gamma) [4], we used the population averaged, stimulus induced instantaneous firing rate as a measure of the spike likelihood in a small interval of time. Through this population averaging of spike trains, intrinsic temporal correlations between spikes are removed. When analyzing the spike trains of individual cells, this population-derived conditional likelihood was used to transform the inhomogeneous point process (i.e. a process with time-varying instantaneous rate) into a homogenous point process with unit rate (Supplementary Fig. 1c, insets)[4, 5]. Note, in the absence of the stimulus, six second long sequences of ISIs were transformed into a point process with unit rate for each cell using the reciprocal of the average ISI obtained from entire baseline recordings (typically 1-2 minutes). The same procedure, outlined below, was applied for the baseline data, simulated Poisson data, and our looming/receding data. The resulting time series allowed us to explore the statistical characteristics of pyramidal cell spiking.

Procedure

The application of the continuous time rescaling theorem[5] has one major drawback for neural data: it relies on the assumption of truly instantaneous events. Since action potentials actually have a 1-2 ms time course, and since data collection/analysis discretizes time, false

rejection of the null hypothesis (H_0 : ON and OFF cell spiking is not significantly different than realizations of a Poisson point process) is inevitable. When applied to our data, we observed the exact same spurious rejection previously reported[4] (see Fig. 1 within reference). To avoid potentially false conclusions that the neurons could not be described as a Poisson process, we applied the more recently published revised methods for discrete time[4]. The steps taken to obtain the rescaled ISIs are explicitly written out in Haslinger et al.[4](see *Section 2.4: Discrete Time Version of Time Rescaling Theorem, Procedure for Analytic Correction*.) After the rate-rescaling step, if the Poisson description of ON and OFF cell spiking is valid, then our empirical ISIs (τ_k) are now exponentially distributed and arise from a homogenous point process with unit rate.

Next, we followed the exact steps outlined in section 2.2 of the original time-rescaling paper[5]. Briefly the rescaled ISIs (τ_k) are further transformed as $z_k = 1 - e^{-\tau_k}$, which yields independent, uniform random variables on the interval (0, 1), assuming the Poisson process description is valid. Next, the z_k are sorted according to length, smallest to largest, and then plotted against the corresponding value of the cumulative distribution function for the uniform distribution, defined as $b_k = \frac{k - 0.5}{n}$ for $k = 1, ..., n$, where n is the total number of ISIs. If the process is indeed Poisson, then the n points should lie along the 45-degree line, contained within the confidence intervals constructed as $b_k + 1.36/\sqrt{n}$ and $b_k - 1.36/\sqrt{n}$, a suitable approximation of the distribution for the Kolmogorov-Smirnov (KS) statistic for a 95% confidence level (α = 0.05). To generate actual *p*-values (included in Fig. 1b and Supplementary Fig. 1b) we performed a more stringent two-way KS test between our transformed ISIs and the uniform distribution (99% confidence, α = 0.01). As an illustration of the method and for the sake of comparison, simulated realizations from a Poisson process (three seconds, the same duration as our in vivo

recordings for v = 2 cm/s) were included (Fig. 1b, Supplementary Fig. 1b): in this case, the null hypothesis that spiking was Poisson could not be rejected. This is an important demonstration, since we are analyzing spiking over small intervals of time (on the order of a few seconds) and thus need to ensure that rejection of the null hypothesis obtained for baseline, looming and receding is not related to an under-sampling of the spiking process (previous studies[4, 5] used very long recording samples).

ISI serial correlation and renewal spiking

The rescaled ISIs further permitted us to examine potential intrinsic temporal correlations (as observed under baseline conditions) of spike timing during stimulation by removing the confounding temporal correlations induced by our looming and receding stimuli (Supplementary Fig. 1c; top, insets). Importantly, the ON and OFF cell population average loses fine temporal information and is a reflection of the stimulus-induced spike likelihood only; thus, any potential history-dependent effects (i.e. memory) in the individual ISI sequences should remain intact after rescaling. The serial correlation was computed for the rescaled ISIs of each stimulus trial and then averaged (Fig. 1c and Supplementary Fig. 1c, bottom). Note the insets, showing the rescaled ISIs with unit rate. The averaged serial correlation function indicates that ON and OFF cell spiking can be treated as a time-inhomogeneous renewal point process (memoryless). This indicates that, for an interval of time $\Delta t = t - t_i$, the chance of observing a spike at time t_{i+1} depends simply on the current value of the stimulus and the timing t_i of the last spike. The renewal assumption is important to the derivation of a relationship to identify the stimulus value that maximizes the Fisher information of non-Poisson rate coding neurons (Supplementary Note 2).

Supplementary Note 2 Application of Fisher Information to Spiking Neurons

The Cramér-Rao bound, the reciprocal of the Fisher information (I_F), is a lower bound on the variance of an observed random variable, in our case the timing of action potentials, conditioned on a stimulus feature (x)[3]. By finding where I_F is maximal along the transverse distance axis ($x \equiv x(t)$), we seek to identify where a decoder of ON and OFF cell firing rates may, in theory, achieve the best possible estimation of changes in object distance from the observed spiking activity.

The I_F of Poisson spiking neurons:

The following is a well-known result, explained without proof in the classic text of Dayan and Abbott[3]. We provide a proof here since the Poisson case is important for understanding our subsequent extension to non-Poisson spiking neurons.

Assume that the discrete probability density (f) of observing n spikes in a given interval of time (Δt) is conditional on the value of a stimulus feature (x) and follows the Poisson distribution:

$$f \equiv \Pr\left[n \middle| x ; \Delta t\right] = \frac{(\lambda(x)\Delta t)^n}{n!} \cdot e^{-\lambda(x)\Delta t}$$

where the rate parameter λ is interpreted as the firing rate. The *score* of the likelihood, with respect to the stimulus feature x, can be determined as follows:

$$
\begin{aligned}
\frac{\partial}{\partial x}\ln[f] &= \frac{\partial}{\partial x}\left(-\lambda(x)\Delta t + n\cdot\left(\ln[\lambda(x)] + \ln[\Delta t]\right) - \ln[n!]\right)\\
&= -\lambda'(x)\Delta t + n\cdot\frac{\lambda'(x)}{\lambda(x)}\\
&= \frac{\lambda'(x)}{\lambda(x)}\cdot\left(n-\lambda(x)\Delta t\right)
\end{aligned}
$$

where $\lambda' \equiv \frac{d}{dx}\lambda$. Starting with the alternative definition of I_F provided in Dayan and Abbott[3] and using $\lambda(x)\Delta t = \mu$, the mean of the Poisson process, we obtain:

$$
\begin{aligned}
I_F &= \left\langle\left(\frac{\partial}{\partial x}\ln[f]\right)^2\right\rangle\\
&= \sum_{n=0}^{\infty} f\cdot\left(\frac{\lambda'(x)}{\lambda(x)}\cdot(n-\lambda(x)\Delta t)\right)^2\\
&= \left(\frac{\lambda'(x)}{\lambda(x)}\right)^2\cdot\sum_{n=0}^{\infty} f\cdot(n-\mu_x)^2\\
&= \left(\frac{\lambda'(x)}{\lambda(x)}\right)^2\cdot\sigma^2
\end{aligned}
$$

where σ^2 is the spike count variance over Δt. Since spiking is assumed to be Poisson here, its spike count variance is equal to its mean spike count in the interval Δt and we have an expression for I_F in terms of the firing rate and its spatial derivative:

$$
\begin{aligned}
I_F &= \left(\frac{\lambda'(x)}{\lambda(x)}\right)^2\cdot\mu\\
&= \left(\frac{\lambda'(x)}{\lambda(x)}\right)^2\cdot\lambda(x)\Delta t\\
&= \frac{\lambda'(x)^2}{\lambda(x)}\Delta t
\end{aligned}
$$

It’s clear that a large change in the firing rate, starting from low frequency spiking, is a more reliable indicator of a stimulus feature than when the same rate of change occurs during high

levels of spiking activity. This expression allows for the direct calculation of the Poisson I_F, referred to in the main text and shown in Supplementary Fig. 2.

Assessing I_F extrema for general spiking statistics:

The goal of the following is to generalize the applicability of I_F to electrophysiological data by ridding ourselves of the Poisson assumption, which is unsatisfactory in many important brain regions displaying irregular spike train statistics. In order to describe general, continuous spiking distributions, we turn to survivor analysis and the hazard rate[2]. The likelihood of observing the ith action potential at time t is a continuous random variable with an event (spike) density function $f(t \mid x) \equiv \Pr[t_i = t \mid x, t_{i-1}]$, conditioned on the time-dependent stimulus feature x (object distance in our study). The associated cumulative distribution $F(t \mid x) \equiv \Pr[t_i \in (t_{i-1}, t_{i-1} + t) \big| x, \{t_n\}_{n=1}^{i-1}]$ gives the probability that the next spike will have occurred by time $t > t_{i-1}$, for a given time course of x and any history-dependent effects in the spiking dynamics generated by the n previous spike times $\{t_n\} \in (-\infty, t_{i-1}]$. The complement of $F(t \mid x)$, the 'survivor' function $S(t \mid x) = 1 - F(t \mid x)$, describes the probability that a spike will not have occurred by time t. The hazard rate, λ, is formally defined as the rate of spike occurrence in a small interval ($\Delta t \rightarrow 0$) and is written as $\lambda(x,t) = \frac{f(t|x)}{S(t|x)}$. This relationship can be well approximated over the short intervals of time (Δt) associated with synaptic transmission, relative to the much longer timescales of motion.

When the probability distributions of ISIs are known, one can use the conditional intensity (the trial averaged instantaneous firing rate) to compute $f(t \mid x)$ directly and then, in turn,

compute the hazard rate[2]. However, the underlying ISI statistics of neurons *in vivo* cannot be characterized by a closed form distribution (e.g. Poisson, Gamma or inverse Gaussian), limiting the applicability of this approach. Furthermore, when determining the conditional event density function we are posed with a serious challenge: smooth motion results in sampling ISIs around instantaneous values of object distance, making it impossible to obtain sufficient numbers of ISIs to properly characterize $f(t \mid x)$ as a continuous distribution. Note that if we were to leave the stimulus at a fixed position, in an attempt to gather large numbers of ISIs, spike-rate adaptation would largely impact the observed patterns of spiking activity and the resulting hazard would not be applicable to natural motion tracking.

To circumnavigate this experimental difficulty, we average the ISIs obtained in many repeated trials as a function of distance, which is the population averaged instantaneous firing rate. In neural spike train analysis, the hazard function for a neuron becomes its theoretical instantaneous firing rate for infinitesimally small intervals[6]. Note that over very small time windows (Δt), the change in position of the stimulus is negligible, and the spiking statistics can be considered stationary with respect to stimulus-induced effects. In other words the timescales associated with spike generation far exceed those of our motion stimuli. This approximation of the hazard rate provides us with a simple and direct connection to our in vivo experiments.

After rate-rescaling the ISIs to remove the stimulus-induced correlations, ON and OFF cell spiking can be characterized as a renewal process, that is, the discharge probability depends only on the current stimulus value and the timing of the last spike (t_{i-1}; Fig. 1c and Supplementary Fig. 1c). This implies that the aforementioned cumulative likelihood, determined over the entire history $(-\infty, t_{i-1})$, is reduced to $F(t \mid x) \equiv \Pr[t_i \in (t_{i-1}, t_{i-1}+t) \mid x, t_{i-1}]$. For some sufficiently short interval $\Delta t = t - t_{i-1}$, for which only one spike should occur, the cumulative

density collapses to the likelihood of observing a single spike, allowing us to approximate it as $F(t \mid x) \cong \Delta t \cdot \lambda(x,t)$, provided Δt is sufficiently small such that $\Delta t \cdot \lambda(x,t) \leq 1 \;\; \forall x, t$. This approximation for small Δt implicitly satisfies the definition of the hazard rate defined in the previous paragraph and is used to simplify the following expression for I_F.

Efron and Johnstone have demonstrated a direct connection between the hazard rate and I_F for arbitrary, real-valued probability distributions[7]. Letting λ_x denote the partial derivative of the hazard rate with respect to x, they showed that

$$I_F = \int_{-\infty}^{\infty} \left[\frac{\lambda_x(x,t)}{\lambda(x,t)} \right]^2 f(t \mid x) dt$$

Their expression holds for all regular conditional probability densities $f(t \mid x)$ on the real line, with corresponding well-defined hazard rates and existing partial derivatives. In order to characterize the ability of a system to encode a stimulus, up until a given moment in time, we assess I_F on the observation interval (- ¥ ,t). Using the approximating relationship between the hazard rate and the survivor function, we see that

$$\begin{aligned} I_F(t) &= \int_{-\infty}^{t} \left[\frac{\lambda_x(x,u)}{\lambda(x,u)} \right]^2 f(u \mid x) du \\ &\cong \int_{-\infty}^{t} \frac{[\lambda_x(x,u)]^2}{\lambda(x,u)} S(u \mid x) du \\ &= \int_{-\infty}^{t} \frac{[\lambda_x(x,u)]^2}{\lambda(x,u)} \big(1 - F(u \mid x)\big) du \\ &\cong \int_{-\infty}^{t} \frac{[\lambda_x(x,u)]^2}{\lambda(x,u)} \big(1 - \Delta t \cdot \lambda(x,u)\big) du \end{aligned}$$

Provided Δt is sufficiently small as required above, the integrand of $I_F(t)$ is a strictly positive function $\forall x, t \in \mathbf{R}$ and unimodal, implying that the value of x for which the integrand is maximal is also where $I_F(t)$ is maximal. Simply put, when searching for the I_F maximum of a neuron's tuning curve, we can locate a focal point by identifying the value of the stimulus for which the integrand achieves a maximal value – in our case, object distance along the transverse body axis [0.25, 6.25] cm. This expression contains the same important relationship found for Poisson spiking but is further modified by the approximated survivor function.

Applying this formula for 1, 2, 3 and 4 cm/s motion yielded clear estimates of the focal point location. As a validation of the new formula, we compared these responses to the relationship for Poisson spiking neurons (Eq. [1]). Supplementary Fig. 2 shows that the Poisson spiking assumption smears the estimate of I_F and makes it difficult to identify one clear maximum. The Poisson formula is relatively more sensitive to slope, which continues to grow rapidly after the onset of bursting and is speed dependent. For example, when applied to 2 cm/s looming data, Eq. [1] still recognizes a local maximum near 1.37 cm but the global maximum on $x \in [0.25, 6.25]$ now occurs at 0.81 cm.

Supplementary Note 3 A simple threshold for burst fraction demarcates the focal point

Bursts are often referred to as "informative" without precise meaning; here we explore their direct contribution to stimulus estimation. Both looming and receding motion are marked by prominent burst spiking, which is noted to occur in the near vicinity of our theoretically identified I_F maximum (Fig. 1a). Thus, we sought to better pinpoint the onset of bursting relative to the focal point, and further test the idea that a downstream decoder could establish a focal point based on a simple bursting criteria.

According to previous work[8] the baseline burst probabilities for ON and OFF cells have a mean and standard deviation of 0.25 +/- 0.014 and 0.22 +/- 0.012 respectively. We started by simply picking a threshold (0.3) significantly greater than these asymptotic estimates (see Fig. 3b) of ON/OFF cell burst fraction (BF). Our reasoning was as follows: first, a BF value of 0.3 infrequently occurs in the ON/OFF cell populations under baseline conditions (Fig. 4c); secondly, extensive bursting across a population of ON or OFF cells could be rapidly detected at this threshold value. With respect to the downstream circuitry, the exact choice of 0.3 is slightly arbitrary but it was inspired by an obvious trend in the data: the burst fraction shoots up after crossing through the focal point region (1.25, 1.45) cm. We looked for a refined BF threshold value that was compatible with our theoretical predictions for 1-4 cm/s (shown in Supplementary Fig. 2), described below.

Burst fraction

In order to determine when bursting became significantly activated, individual spike trains were separated into tonic (ISI > 10 ms) or burst (3 < ISI < 10 ms) spikes[9]. Burst fraction is computed as the number of burst spikes, divided by the total number of combined tonic and burst spikes. In previous work, BF was either determined in the absence of a stimulus or during presentation of stationary signals (e.g. sinusoidal EOD amplitude modulations), and thus the proportion of burst spikes was determined over the entire duration of a recording (on the order of seconds to minutes); for obvious reasons (see Fig. 3b) we refer to this as the asymptotic BF. The estimates of our population asymptotic BF were determined from entire baseline recordings for each cell (ΔT = 60-120 s) and yielded a mean and standard deviation of 0.15 +/- 0.11 for ON

cells and 0.22 +/- 0.12 for OFF cells, consistent with in vitro measurements for the centrolateral map of the ELL[10] and reflecting a good representative sample of ELL pyramidal cells.

For non-stationary ON/OFF cell responses to motion, we are dealing with a BF conditioned on a time-dependent stimulus; therefore we must count spikes within small intervals of distance (Δx). For our different experimental conditions, this resulted in different time windows (each one calculated as $\Delta T = \Delta x / |v|$, where v is object velocity). Figure 3b shows that the estimated baseline BF changes as a function of the time window. Not surprisingly, as $\Delta T \rightarrow 0$ the chance of observing a burst event (i.e. multiple spikes) becomes very unlikely and the BF rapidly becomes underestimated compared to the asymptotic estimates. Therefore, when choosing a spatial interval, there is a trade-off to consider. A small Δx gives great spatial resolution but moves ΔT into a range that grossly underestimates the BF. The other extreme is a large ΔT, such that even for the 4 cm/s looming stimulus the estimated BF is somewhat near its asymptotic value (Fig. 3b). This results in poor spatial resolution and quickly begins to defeat the purpose of our analysis – precisely determining the location of an FI maxiumum. For Figures 3b, 3c and 4b in the main text, $\Delta x = 2$ mm was chosen as the minimal interval that, when divided by our top speed, gave an acceptable ΔT while maintaining good spatial resolution. As shown in Supplementary Table 1, $\Delta x = 2$ mm gave results that were very compatible with Eqs. [1] and [2], whereas shorter intervals of 1 and 1.5 mm were less reliable. However, when in agreement with the theory, these shorter spatial intervals were useful as they allowed us to compute the intersection of the different Δx and obtain improved spatial resolution for the identification of the focal point.

Note that $\Delta x = 2$ mm is a reasonable spatial resolution given the standard deviation for the position of optimal behavioral performance (1.7 mm; Fig. 2b). For each time window ΔT,

used in our analysis, a population BF was computed for each of the populations associated with the eight different stimulus conditions, using the first (last) 2 cm of the looming (receding) stimuli, where there are no discernible stimulus effects. These different BF values were then averaged giving a population average BF and standard deviation as a function of the time window. This average value was used to align each stimulus condition's population BF, so that it could be accurately compared with the other cases. This counteracts the drift due to different windows of time associated with each stimulus condition and the fixed spatial interval, as well as any small discrepancies based on the slightly different, though largely overlapping, ON/OFF cell populations obtained for our various stimulus conditions. Our hard threshold of 0.3 was given a small buffer zone of +/- 0.005 for cases, as in a few cases BF values were practically on 0.3. In a concerted effort not to overestimate the BF, we did not adjust the baseline mean of the stimulus condition if it was a standard deviation (ranging from 0.02-0.04 as a function of ΔT) below the population mean from all conditions for that particular time window. This is important because the dependence of the BF estimate on the time window is further influenced by the stimulus distance and the degree of burst activation. In the more activated state, BF is less susceptible to underestimation. This can result in inflated values of the BF near the focal point.

The burst fraction plots for the different stimulus conditions are presented in Figs. 3c and 4b. Note that the 24th BF interval spans from 1.25 to 1.45 cm along the distance axis. We found that the 24th interval is consistently just shy of the threshold and that from the 25th interval onward, there are substantial, speed-dependent increases in the BF. From the figures it is clear that bursting activity is increasing and is detectable in the 24th interval, but clearly the majority of the population has not fully transitioned to the burst state (Fig. 4c). Based on our improved Fisher information criterion, this is where we find x^*, indicating that optimal estimation is

achieved if the animal can maintain a distance near the location of a bifurcation to bursting in the population, where approximately half the units have transitioned to bursting (Fig. 4c).

Our simple burst criterion is used as a means of assessing the relationship between bursting and optimal stimulus estimation, in addition to extending our analysis to stimulus conditions that cause significantly weaker firing rates (slower speeds or smaller spheres), where the theoretical analysis becomes less practical. This BF threshold of 0.3 was chosen based on our particular sample of the ON/OFF cell population under study and the choice of the burst fraction interval. In reality, we expect that downstream synapses in the midbrain are adapted to baseline burst statistics for a given decoding timescale (Δt) and that, unlike the hard-threshold used in our analysis, a soft dynamic threshold is more likely utilized in freely swimming fish. In addition to BF, encoding BF slope is also likely important. Stimulus intensity could be encoded as relative changes in BF, where the tonic and burst spikes are extracted by facilitating and depressing synaptic dynamics[11, 12]. However, these speculative ideas will require extensive further study and are beyond the scope of this paper.

Supplementary References

1. Shimazaki, H. & Shinomoto, S. A method for selecting the bin size of a time histogram. *Neural Comput.* **19**, 1503-1527 (2007).

2. Barbieri, R., Quirk, M.C., Frank, L.M., Wilson, M.A. & Brown, E.N. Construction and analysis of non-Poisson stimulus-response models of neural spiking activity. *J. Neurosci. Meth.* **105**, 25-37 (2001).

3. Dayan, P. & Abbott, L.F. *Theoretical neuroscience: computational and mathematical modeling of neural systems* (MIT Press, Cambridge, MA, 2001).

4. Haslinger, R., Pipa, G. & Brown, E. Discrete time rescaling theorem: determining goodness of fit for discrete time statistical models of neural spiking. *Neural Comput.* **22**, 2477-2506 (2010).

5. Brown, E.N., Barbieri, R., Ventura, V., Kass, R.E. & Frank, L.M. The time-rescaling theorem and its application to neural spike train data analysis. *Neural Comput.* **14**, 325-346 (2002).

6. Kass, R.E., Eden, U.T. & Brown, E.N. *Analysis of neural data* (Springer-Verlag, New York, NY 2014).

7. Efron, B. & Johnstone, I.M. Fisher's information in terms of the hazard rate. *Ann. Statist.* **18**, 38-62 (1990).

8. Bastian, J. & Nguyenkim, J. Dendritic modulation of burst-like firing in sensory neurons. *J. Neurophysiol.* **85**, 10-22 (2001).

9. Clarke, S.E., Longtin, A. & Maler, L. A neural code for looming and receding motion is distributed over a population of electrosensory ON and OFF contrast cells. *J. Neurosci.* **34**, 5583-5594 (2014).

10. Mehaffey, W.H., Maler, L. & Turner, R.W. Intrinsic frequency tuning in ELL pyramidal cells varies across electrosensory maps. *J. Neurophysiol.* **99**, 2641-2655 (2008).

11. Middleton, J., Yu, N., Longtin, A. & Maler, L. Routing the flow of sensory signals using plastic responses to bursts and isolated spikes: experiment and theory. *J. Neurosci*. **31**, 2461-2473 (2011).

12. Fortune, E.S. & Rose, G.J. Short-term synaptic plasticity contributes to the temporal filtering of electrosensory information. *J. Neurosci.* **20**, 7122-7130 (2000).
